# Who takes the lead in oral surgery simulation? Students’ perceptions and practical skills towards virtual reality and phantom model training: a comparative study

**DOI:** 10.1515/iss-2025-0008

**Published:** 2025-06-26

**Authors:** Juliane Kröplin, Constanze Veronika Friedrich, Lisa Harms, Ingo Buttchereit, Jan-Hendrik Lenz, Bernhard Frerich

**Affiliations:** Department of Oral and Maxillofacial Surgery, Facial Plastic Surgery, University Medical Centre Rostock, Rostock, Germany; Office of Medical Education, University Medical Centre Rostock, Rostock, Germany

**Keywords:** virtual reality, simulation training, oral surgery, sustainability, serious games, oral – maxillofacial surgery

## Abstract

**Objective:**

Virtual reality (VR) simulators are considered a promising tool for significantly enhancing surgical skills. This study aims to compare the use of a VR simulator with a conventional phantom model in oral surgery training. The findings should contribute to the further development of oral surgery simulation training and help maximize the innovative benefits of VR-based training.

**Methods:**

This prospective, cross-sectional, single center study analyzed students’ competence in performing an osteotomy on a displaced tooth 38. Participants were dental students in their 5th semester at a University Medical Center in Germany. The study compared a VR simulator with a phantom model. After completing the exercise, students filled out a questionnaire consisting of both standardized and open-ended questions to allow for a comprehensive subjective evaluation of the training experience.

**Results:**

A total of 22 students took part in the study (12 female, 10 male). None had previously performed the exercise on a patient. From the students’ perspective, both training methods were easy to learn (VR:2.0; Ph: 2.1; p=0.642). Individual feedback highlighted that the VR simulator’s main advantages were sustainability (n=12) and objectivity (n=5). However, standardized responses indicated that the phantom model was rated as more realistic, particularly regarding anatomical representation (p<0.001), haptic feedback (p<0.001) tooth removal (p<0.001) and osteotomy steps (p<0.001). Both exercises were equally engaging for students (VR:1.8; Ph:1.5; p=0.162).

**Conclusion:**

Simulation-based training is an engaging way for students to learn surgical tooth removal. Currently, conventional simulation is subjectively perceived as superior to VR simulation, particularly in terms of realism. However, VR simulation allows for a more objective assessment of performance and is considered superior to the phantom model in terms of sustainability. Further technological advancements and improvements in realism could help maximize the innovative benefits of VR-based training.

## Introduction

The educational benefits of simulation-based teaching are well-documented in the literature [1], 2]. Additionally, rapid technological advancements continue to enhance virtual simulation systems [3], 4]. The 2020 amendment to Germany’s dental licensing regulations has also necessitated a shift of dental education [5]. While digitalization is transforming various sectors of society, the economy, and the workplace, higher education often lags behind these developments [6].

Despite the growing digitization in dentistry, such as the introduction of digital impressions with intraoral scanners and the use of CAD/CAM technology for digital planning and manufacturing, the actual dental education remains largely non-digital [7]. Traditionally, dental education involves hands-on exercises using physical models, such as caries preparation and surgical training [8]. The frequent use of plastic models for practice results in ongoing material costs, placing a financial burden on students. This limits their opportunities to practice, exacerbating social inequalities, and leads to subjective performance evaluations, further contributing to disparities [7], 8].

Virtual reality (VR) is emerging as an innovative teaching tool that offers a different approach compared to conventional plastic models. VR simulators allow students to practice procedures repeatedly without incurring additional material costs – only requiring time [9]. This reduces financial barriers associated with conventional training methods, creating a more accessible and equitable learning environment. Additionally, VR technology enables objective assessment by digitally evaluating and reviewing student performance. Particularly during the pandemic, when patient care and student courses with real patients were suspended, the need for technological and digital solutions became more pronounced [10]. VR can provide substantial value by supporting routine procedures and potentially integrating with other applications, such as virtual patients that simulate pain and anxiety, thus enhancing both practical skills and students’ emotional preparedness [11].

Despite its potential, VR technology still faces significant challenges, including technical limitations and high initial costs. However, it offers the flexibility to accommodate different learning styles while bridging the gap between theory and practice [12]. VR can also enhance fine motor skills and hand-eye coordination by providing an immersive, hands-on training experience [13]. Future developments in VR simulations could offer even more realistic training scenarios, accurately replicating intraoral interactions, patient positioning, and the handling of essential tools such as drills, mouth mirrors, and suction devices. These advancements may also include sensory feedback, as well as simulations of various tooth positions and anatomical anomalies [14].

While the potential of VR in dental and oral health education is promising, research on its effectiveness remains limited [11]. It is essential to critically assess not only the simulations themselves but also the broader learning process – including preparation, briefing, feedback, debriefing, reflection, and evaluation [15].

This study aims to compare the use of a traditional physical model with the expanded capabilities of VR in oral surgery training. It seeks to explore the potential added value of VR, identify the technical and pedagogical challenges, and scientifically test these new teaching methods for integration into educational curricula [13].

## Methods

### Participants

A total of 22 students participated in the study. Participants were dental students from a University Medical Centre in North Germany in their 5th semester in the winter semester 2023/24. The students were the first cohort to be trained under Germany’s new dental licensing regulations. The exercise was carried out as part of compulsory teaching. Exclusion criterion was the wish not to participate in the semester. However, this did not affect any of the participants.

### Study structure and design

**Figure 1:**
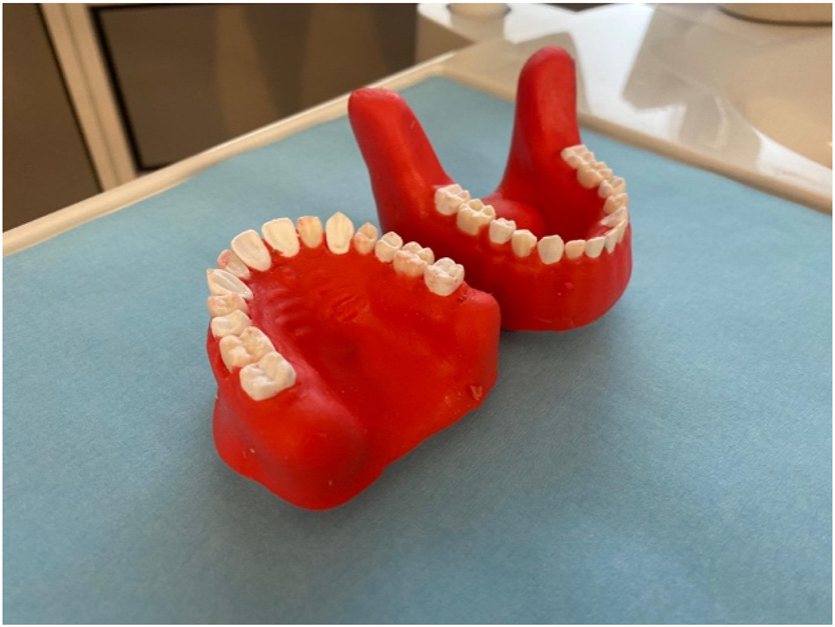
Mednerva oral and maxillofacial surgical training model (maxilla and mandible).

**Figure 2:**
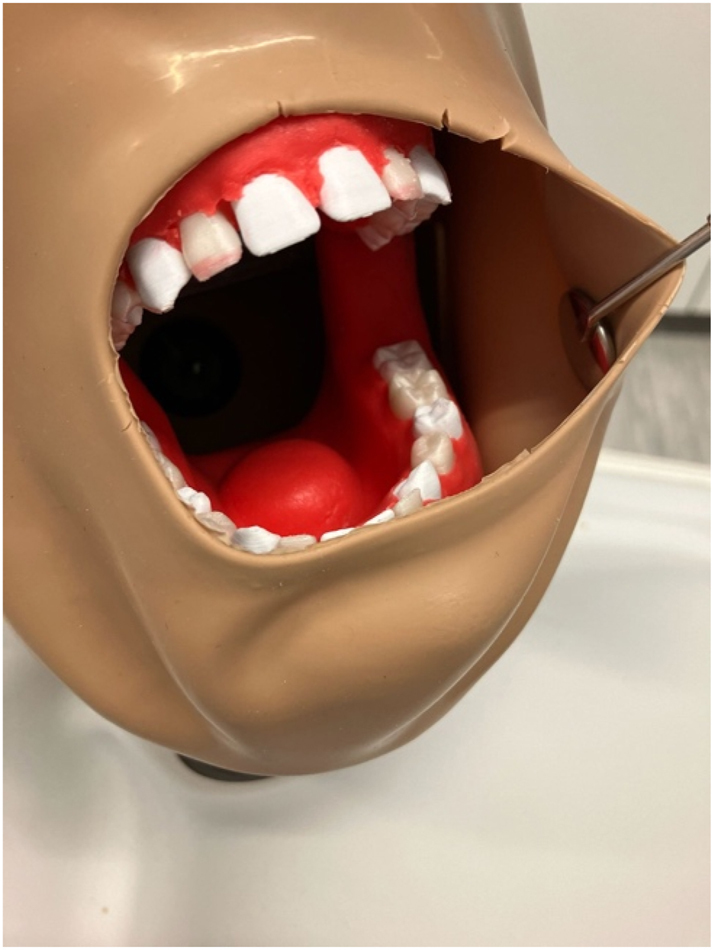
Mednerva oral and maxillofacial surgical training model in phantom head.

**Figure 3:**
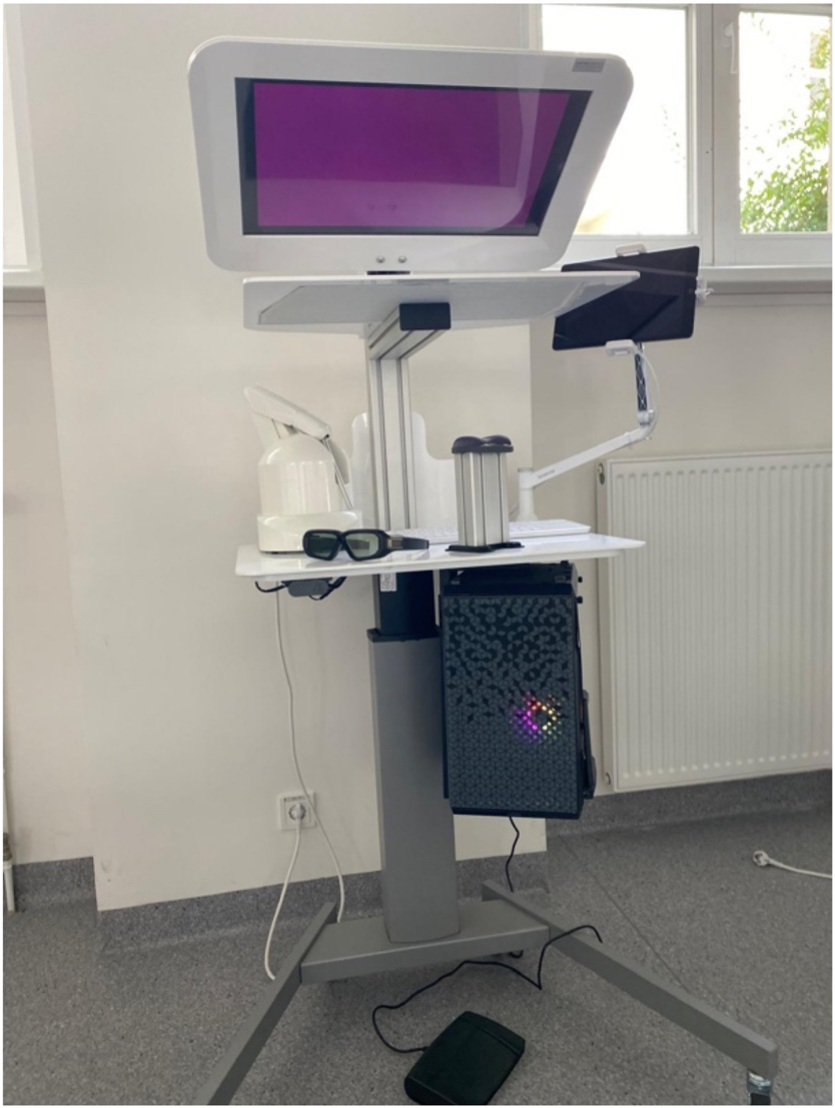
Depiction of the KOBRA simulator hardware.

This study was designed as a prospective, cross-sectional, single center study. It was meticulously planned and approved by the Ethics Committee of the University of Rostock, ensuring compliance with all ethical guidelines (Reference number: A 2023-0178). This study aimed to compare the acceptance and effectiveness of a conventional simulator (KaVo simulation unit equipped with Mednerva oral and maxillofacial surgical training model “Ph”) and a virtual reality simulator (KOBRA simulator, “VR”) for performing an osteotomy on tooth 38. To optimize supervision, participants were divided into two groups, which were further subdivided into smaller teams so that both simulators could be used simultaneously. In the first session, all students received hands-on guidance from the study leader on how to use the phantom model. In the second session, they were given a similar introduction and practice time with the VR simulator. Each introduction lasted 30 min and was delivered as an oral presentation, followed by a demonstration on the respective simulator. Some students started with the phantom model (n=12, Figures 1 and 2) [16], while others began with the VR simulator (n=10, Figure 3) [17]. All students utilized both simulators. Due to time and personnel resources within the framework of compulsory teaching, it was not possible for all students to start with the same simulator.

### Data collection

After each exercise round, students completed a custom-designed questionnaire (Table 1) that included both standardized and open-ended questions. This allowed for a thorough subjective evaluation of the exercise. The standardized questions were rated on a Likert Scale, while the open questions provided in-depth qualitative feedback. The categories were defined by two researchers. The frequency of the categories mentioned in the open questions were counted and documented. Additionally, one instructor per simulator conducted an objective assessment of the exercise results (Table 2). For this, a standardized questionnaire with five-point scale was used to ensure a detailed evaluation of performance.

**Table 1:** The questionnaire for the subjective assessment of the participating students.

Question about previous experience
Have you already performed the respective exercise before this course? – Yes, in a simulation exercise. – Yes, on patients. – No

**Open questions**

What advantages do you see in the VR simulator compared to the phantom model?
What advantages do you see in the phantom model compared to the VR simulator?
Which improvements would you suggest for the exercises on the phantom model?
Which improvements would you suggest for the exercises on the VR simulator?

**Standardized questions for evaluation on a Likert scale from 1 to 5 (1=fully applies - 5=does not apply at all)**

1. The anatomical representation is realistic.
2. The haptic feedback is realistic.
3. The simulation of the osteotomy is realistic.
4. The simulation of the tooth extraction is realistic.
5. It was easy for me to learn the given exercise.
6. I would have liked more practice time. If you selected 1–2, how much additional time would you have wished for?
7. Through the simulation training, I was able to improve my practical skills.
8. Through the simulation exercise, I feel well prepared for the practical application on patients.
9. The exercise was engaging.
10. I Would like to have more training with the phantom simulator/VR simulator.

**Table 2:** Evaluation parameters of the practical examination performance.

Evaluation of the examination performance
Required time:
*Evaluation criteria with a rating scale from very good (1) to insufficient (5) or not applicable*
Anesthesia
Surgical exposure of the crown
Tooth extraction
Extent of osteotomy/iatrogenic bone loss
Damage to adjacent teeth
Wound management

### Exercise on phantom-simulator

Figures 1 and 2 present different perspectives of the Mednerva oral and maxillofacial surgical training model [16]. The exercise on the phantom simulator means that each tooth can only be osteotomized once, because the tooth is surgically removed by osteotomy. To ensure comparability between the different simulators, tooth 38 had to be saved for the examination, as each student only had one conventional model available. Instead, practice was conducted on tooth 18 (Figure 2).

### Exercise on VR-simulator

The simulator used is the KOBRA Simulator. This is a commercial VR simulator that is distributed via the internet platform kobra.habtikenfrabriken.com. The simulator also offers other learning tools such as an apicoectomy or the removal of other displaced teeth.

**Figure 4:**
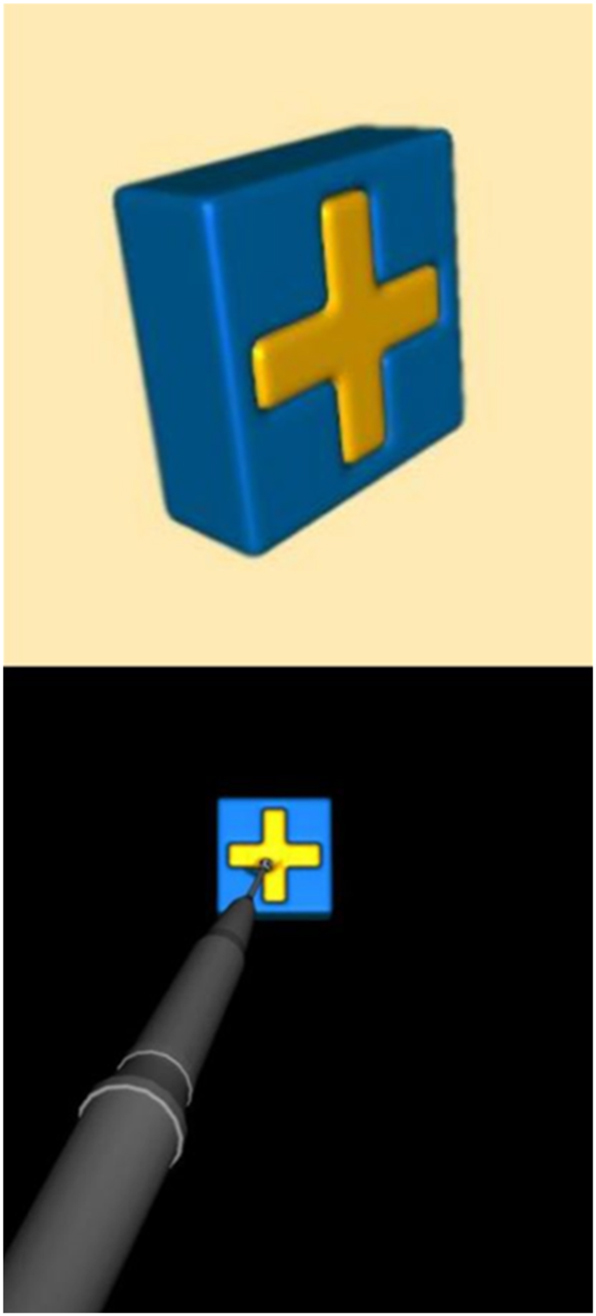
Illustration of the crossbox training programme on the virtual reality simulator; with (top) and without (bottom) handpiece.

**Figure 5:**
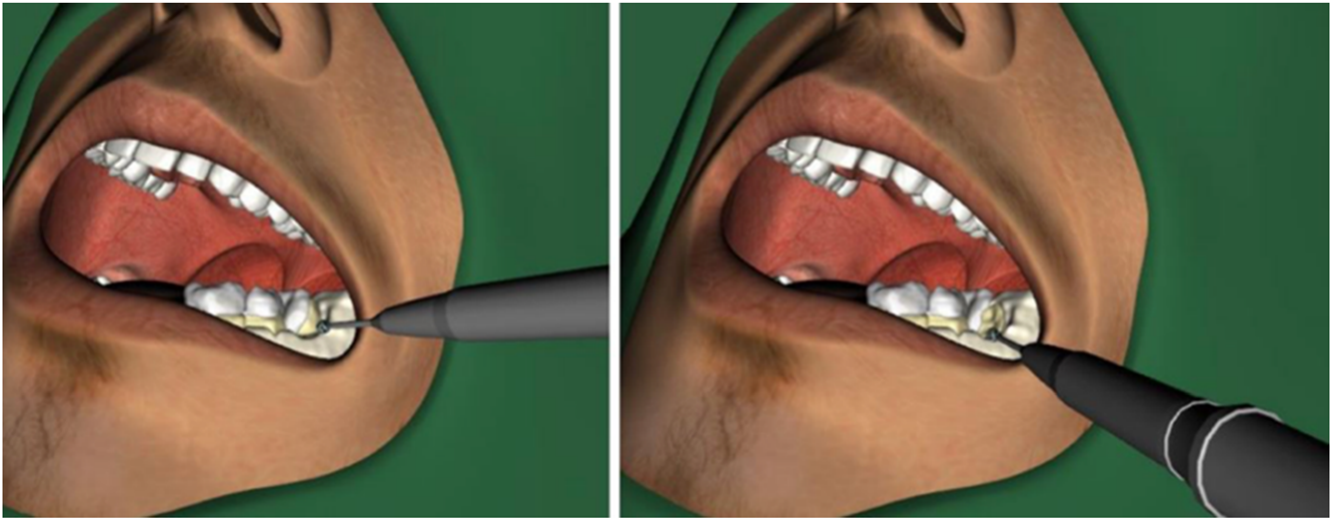
View of the oral section of the patient in the VR simulator and illustration of two different steps in the osteotomy procedure for tooth 38.

The VR training unit, as shown in Figure 3, consists of a height-adjustable training system with 3D glasses. The simulator is operated using a handpiece that replicates the hand movements required for an osteotomy on a patient. The procedure is projected onto a screen, and the 3D glasses allow users to view the movements in three dimensions. To familiarize themselves with the VR simulator, all students had the opportunity to complete a practice exercise, as shown in Figure 4 [17]. This exercise involved removing a yellow cross, simulating the haptic feedback of an osteotomy. The goal was to remove the yellow cross without damaging the surrounding blue box. Following this, students proceeded with the surgical removal of tooth 38, as illustrated in Figure 5 [17]. Depending on their individual skill level, speed, and understanding of the procedure, students were able to complete between two and four practice sessions within the allotted one-hour training period. The VR simulator was used exclusively for practicing the osteotomy of tooth 38. During the training, the parameters ‘Jawbone’ (iatrogenic bone loss), ‘Lingual Bone’ (iatrogenic bone loss of the lingual bone), and ‘Tooth 7’ (injury to the neighboring tooth) were recorded in cubic centimeters (cm^3^) and visualized on a tablet.

To gain a comprehensive understanding of the effectiveness and acceptance of the two training methods, we compared students’ subjective assessments with instructors’ evaluations of exam performance. By combining standardized and open-ended questions, we were able to conduct a detailed analysis of the advantages and disadvantages of VR simulation compared to traditional phantom simulation.

### Evaluation of students’ examination performance

The students’ examination performance was assessed based on their execution of the osteotomy on tooth 38. The evaluation criteria are outlined in Table 2. Since anesthesia and wound care could not be performed using the VR simulator, these aspects were not included in the evaluation. Objective data from the VR simulator were utilized to evaluate specific sub-steps, including the extent of the osteotomy and any damage to adjacent teeth. These parameters were recorded during the exercise and visualized on a tablet. The extent of the osteotomy was assessed through peer comparison using the parameters loss of jawbone and loss of lingual bone which were collected by the simulator and visualized on the iPad. Both the mean and median values of these parameters were calculated. Examination performance was then categorized into quintiles (<Q_0,2_=1; <Q_0,4_=2; <Q_0,6_=3; <Q_0,8_=4; >Q_0,8_=5). The evaluation of the crown visualization sub-step was determined by the number of additional osteotomies required before successful tooth removal. Students presented their surgical result to the examiner before using the elevator, once they deemed the osteotomy sufficient. If the tooth could not be removed successfully, they were allowed to repeat the procedure until removal was achieved (no additional osteotomy=1, up to four additional osteotomies=5). Tooth removal was assessed based on the number of assists requested by students from the examiners (no assists=1, up to four assists=5). Assistance was provided only upon the student’s request. The examiners were specialists in oral surgery and therefore qualified to assess the performance of the exercise. The time was measured using two standardized clock: one for each simulator. There were two instructors in total, each assigned to a simulator.

### Statistical analysis

The data was analyzed using IBM SPSS v27 (Armonk, N·Y., USA) software. The gender distribution, previous experience and the subjective und objective assessment were analyzed descriptively. Statistical analysis of phantom vs. VR simulation was performed by an unpaired, two-tailed Student’s *t*-test. A value of p<0.05 was considered statistically significant.

## Results

### Participants

A total of 22 students participated in the course (12 female; 10 male). None had previously performed the exercise on a patient. None had previously carried out the exercise on the KOBRA virtual reality simulator. Which simulator was started with had no measurable influence on the results.

### Subjective assessment – standardized questions

Table 3 presents the results of the subjective assessment. With the exception of the aspect of engagement (Q9) and learnability (Q5), the phantom model was found to be significantly superior overall.

**Table 3:** Results of the subjective assessment rated on a Likert scale from 1 to 5.

Question	Phantom sim mean (std. deviation)	VR sim mean (std. deviation)	p-Value
All	1.9 (0.9)	3.1 (1.1)	<0.001
Anatomical representation (Q1)	1.9 (0.8)	2.8 (1.1)	0.002
Haptic feedback (Q2)	2.1 (0.9)	3.4 (1.0)	<0.001
Osteotomy (Q3)	1.8 (0.9)	3.1 (1.0)	<0.001
Tooth extraction (Q4)	1.9 (0.9)	3.8 (1.0)	<0.001
Learning experience (Q5)	2.1 (1.0)	2.0 (1.2)	0.642
More practice (Q6)	2.2 (1.0)	3.6 (1.4)	<0.001
Improved skills (Q7)	1.6 (1.1)	3.5 (1.0)	<0.001
Prepared for patients (Q8)	2.7 (1.0)	3.9 (1.0)	<0.001
Engaging (Q9)	1.5 (1.0)	1.8 (0.9)	0.162
More training (Q10)	1.6 (1.1)	2.9 (1.3)	<0.001

### Subjective assessment – open questions

**Figure 6:**
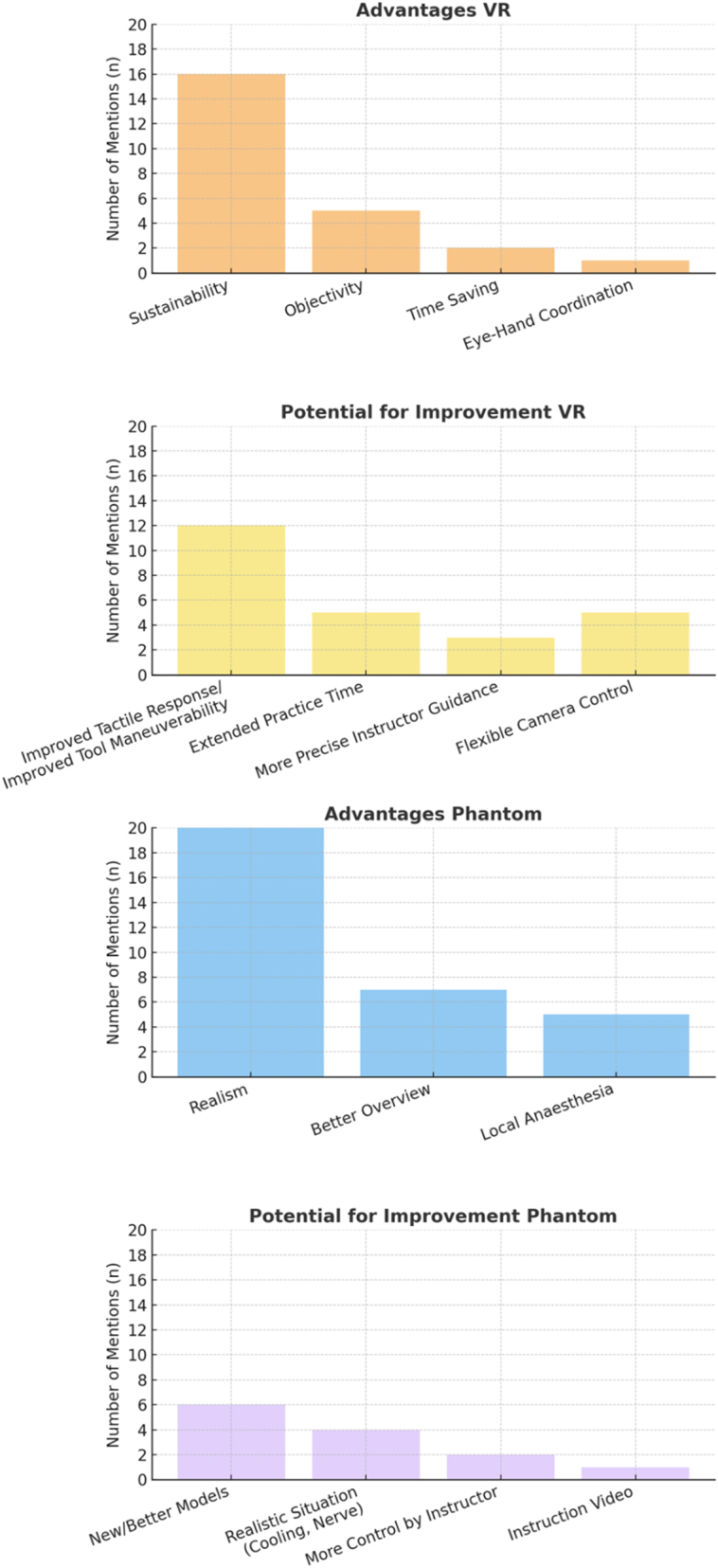
Overview of advantages and potential for improvements for both simulators. The advantages of VR simulation are primarily found in its objectivity and sustainability. The phantom model is regarded as more realistic.

The subjective qualitative analysis is presented in Figure 6. From the students’ perspective, the advantages of VR simulation are primarily found in its objective evaluation of results and its sustainability. The phantom model is regarded as more realistic.

### Evaluation of students’ examination performance

Table 4 presents the results of the evaluation by the study supervisors. The students required significantly less time to complete the exam on the VR simulator. No significant gender-specific differences were observed in the exam results on either the phantom or VR simulator. Due to the differing assessment methods, a direct comparison of performance between the VR and phantom simulators cannot be drawn. Table 5 presents the quintiles for determining the extent of osteotomy on the VR simulator.

**Table 4:** Results of the assessment by the study supervisors rated on a Likert-scale from 1 to 5 and the time required for the examination.

Sub-steps examination performance by study supervisors	Results phantom mean (std. deviation)	Results VR mean (std. deviation)	p-Value
Anesthesia	1.6 (0.6)	Not to be assessed	–
Surgical exposure of the crown	1.9 (0.7)	2.4 (1.5)	0.18
Tooth removal	1.8 (0.8)	1.2 (0.4)	0.01
Extent of osteotomy/iatrogenic bone loss	2.1 (0.6)	2.7 (1.5)	0.04
Wound care	1.5 (0.5)	Not to be assessed	–
Damage to adjacent teeth	Yes: n=1 No=n=21	Yes: n=6 No: n=21	0.02

**Time required for the examination**	**Results phantom** **minutes (std. deviation)**	**Results VR** **minutes (std. deviation)**	**p-Value**

Time	35.6 min (8.0 min)	2.5 min (1.9 min)	<0.001

**Table 5:** Quantification of the removed bone in the virtual reality simulator.

Extent of osteotomy	Q_0,2_ mm^3^ (n=*x*) =1	Q_0,4_ mm^3^ (n=*x*) =2	Q_0,6_ mm^3^ (n=*x*) =3	Q_0,8_ mm^3^ (n=*x*) =4	Q1 mm^3^ (n=*x*) =5	Mean (median)
Jawbone	117.5 (n=1)	235.0 (n=9)	352.4 (n=3)	469.9 (n=6)	587.4 (n=3)	3.1 (2.0)
Lingual bone	1.6 (n=12)	3.3 (n=3)	4.9 (n=0)	6.6 (n=3)	8.2 (n=4)	2.3 (2.0)

## Discussion

### Demographics

With the introduction of oral surgery simulation training into our faculty’s dental curriculum in the winter semester of 2023/2024, all 5th-term dental students had the opportunity to practice the surgical removal of wisdom teeth. The implementation of this new teaching format was aligned with the updated dental licensing regulations, which emphasize innovative educational objectives, including training in evidence-based evaluation of medical and dental procedures [5]. By reflecting on the advantages and potentials of improvement of both systems, students are encouraged to critically engage with innovative teaching concepts, ultimately contributing to their further development.

### Subjective assessment by students – summary of findings and current literature

In terms of subjective assessment, the phantom model was rated superior to VR simulation training in almost all aspects, particularly in terms of realism. A review of the current literature reveals that there are very few prospective randomized studies evaluating the impact of oral surgery simulator training. VR technology is most commonly used in implantology and orthognathic surgery [18]. Existing studies on simulated oral surgery procedures primarily showcase the capabilities of VR technology but do not provide a direct comparison with conventional simulation methods [3], 14].

A review by Towers et al. on the use of VR in preclinical education highlighted the lack of established standards for dental simulators. Most available dental simulators have not been validated [19]. Additionally, most studies focus primarily on technical skills. In this context, Ayoub et al. emphasize that future research should take a more holistic approach to learning – incorporating not only technical skills but also non-technical skills such as teamwork, interpersonal communication, and cognitive development [18]. Furthermore, it has been suggested that integrating artificial intelligence could enhance interprofessional collaboration [20]. Although students completed the VR simulator exercises in groups, the subjective assessment did not include specific feedback on teamwork. However, observations during the exercises indicated that students actively communicated with one another – mainly by offering assistance and sharing their own experiences to support those performing the exercises. Nonetheless, based on the current results, it is unclear to what extent collaboration improved practical performance. Future studies should consider a formal assessment of teamwork and faculty feedback.

Interestingly, none of the students mentioned financial considerations. This is surprising given that the VR simulator is provided by the clinic, whereas the conventional model is not. As a result, students do not incur additional costs when training on the VR simulator, whereas with the traditional model, they must purchase the training model and some consumable materials themselves. From a student’s perspective, the VR simulator offers both ecological and economic advantages. For our institute, however, the initial acquisition costs were substantial. In the long term, cost savings may arise from reduced personnel expenses, as the simulator independently assesses performance. Additionally, having a VR simulator could provide a competitive edge in attracting top faculty members, students, and residents.

### Students’ examination performance – summary of findings and current literature

In a 2022 pilot study by Ming-Yi et al., a total of 16 interns tested the KOBRA simulator as well. The results showed that the subjective learning effect of performing a surgical extraction of tooth 38 was rated relatively low compared to other aspects – such as improved hand-eye coordination, clinical confidence, and environmental sustainability [3]. The authors attributed this to the lack of simulation for anesthesia, incision techniques, flap reflection, bleeding, and wound closure in the tested model – an aspect of VR simulation that was also criticized by our participants. Enhancing the software to include soft tissue management training could therefore improve user satisfaction and increase the learning effect. However, focusing solely on osteotomy and tooth extraction in the VR simulator can also be seen as an advantage, as it allows students to concentrate on these specific steps. This is particularly beneficial for inexperienced students, enabling them to refine their hand-eye coordination without being overwhelmed by the complexity of the entire procedure.

Several studies have examined the role of virtual simulation-based dental education in achieving the United Nations’ Sustainable Development Goals (SDGs) [3], 21], 22]. In particular, virtual simulation is seen as a valuable tool in supporting three key SDGs: ‘Quality Education’ (SDG 4), ‘Reduced Inequalities’ (SDG 10), and ‘Responsible Consumption and Production’ (SDG 12). This highlights the significant role of digital transformation in education from a healthcare policy perspective, a sentiment that was also reflected in our students’ feedback.

The perspective of course instructors was not surveyed in this study. However, certain aspects are particularly relevant to the SDGs ‘Quality Education’ (SDG 4) and ‘Reduced Inequalities’ (SDG 10). For instance, VR simulation allows instructors to minimize unconscious bias by relying on objective assessment criteria. Furthermore, students practiced more independently after receiving initial instructions, as they could review their own performance without relying solely on instructor evaluations.

When analyzing the examination results, the first striking observation is that performing the examination on the phantom simulator takes significantly longer. This can largely be attributed to the broader treatment scope of anesthesia and soft tissue management involved in the conventional simulator. These sub-steps were not assessed separately in the VR simulator. Additionally, the available literature does not provide validated evaluation standards.

The evaluation criteria we selected (Table 5) allow for objective evaluation in the VR simulation for the sub-steps ‘Damage to adjacent teeth’ and ‘Extent of osteotomy’ by visually capturing performance. Partial objectification was also possible for the ‘Tooth removal’ sub-step. However, when interpreting the test results, it is important to consider that the two systems are not fully comparable. By using quintiles, it was at least possible to compare the groups within the VR training unit, taking objective data into account. A direct comparison within the VR simulator itself allows for an objective analysis of system-generated data. Buchbender et al. followed a similar approach in their case-control study [14]. Our chosen evaluation method therefore represents a compromise aimed at achieving comparability between conventional simulators and VR simulation, in line with the study’s objectives.

Regardless, the ability to objectively assess examination performance in the VR simulator, compared to the conventional model, should be highlighted. This was not only positively received by students but also aligns with the UN Sustainable Development Goal ‘Reduced Inequalities.’ By minimizing limitations due to unconscious bias and inter-rater variability, this approach has the potential to improve examination fairness. It must also be taken into account that the time and personnel resources available for teaching are limited. As a result, peer assessments by several examiners are often not possible.

Furthermore, the ability to measure performance enables students to self-evaluate and continuously improve – even without direct instructor supervision [23]. This directly supports the new ZAppprO training objective of ‘Qualification for initial and continuing education.’ At the same time, it offers a potential solution to the ongoing shortage of specialized staff without compromising teaching quality. The benefits of instructor-independent practice have been previously described [24]. Expanding on this concept, there is ongoing discussion about examinations that are independent of instructors as well. The objective documentation of examination performance in VR training enables assessments to be conducted independent of time and location, representing a key advantage over the phantom model.

### Perspectives in digital oral surgery simulation

The current findings indicate that VR training cannot yet fully replace conventional simulation without limitations. However, in the long term, further development of existing technology is certainly worthwhile. The key advantages of VR training – such as its sustainability and the objectivity of training and evaluation – support this rationale. To address the lack of realism noted by students, technical enhancements, for e.g. soft tissue handling and anesthesia simulation, might narrow the perceived gap between VR and conventional simulation. Additionally, integrating augmented reality (AR) could be a promising way for further technical advancements [20]. AR allows digital simulation training to be projected into the real world [23] enhancing the sense of realism. Additionally, artificial intelligence (AI) holds great potential for user-centered, continuous development by processing ever-growing amounts of data [25], 26]. In the context of VR and AR simulation training, big data analytics enables the mapping of real-world complexity, thereby optimizing skill acquisition. Personalized feedback can further support decision-making processes, ultimately improving treatment quality and patient safety [20].

The principal advantage of objective data collection lies in its potential to standardize examination performance across institutions and locations. This, in turn, would facilitate comprehensive comparability of students’ competencies and promote the further development of teaching that is responsive to individual learning needs.

Another promising aspect is that students found the simulation training engaging in both formats, highlighting opportunities for incorporating a gamification approach. Gamification involves integrating game-like elements into a serious learning context, a method that has been shown to enhance learning outcomes [27]. Additionally, research suggests that regular video gaming can positively impact digital simulation training. For instance, Rosser et al. identified a positive correlation between frequent gaming and improved laparoscopic skills [28]. However, a comparable effect on oral surgery skills using a VR simulator has not yet been reported. Since our study did not collect data on this aspect, we cannot make any definitive claims. Nevertheless, based on instructional feedback, our findings, and existing literature, it is evident that students are still relatively unfamiliar with digital teaching and learning tools [20]. Our results underscore the need for a more digitalized approach to education, emphasizing the implementation of innovative teaching and learning methods.

## Limitations

The main limitations of this study include its single-center design and the small sample size of students. Another limitation is the lack of a comprehensive analysis of learning outcomes beyond subjective perceptions and task completion rates. Moreover, the study focuses exclusively on osteotomy training. Expanding the scope to include other surgical skills could enhance the generalizability of the results. Further prospective studies are needed to specifically address objective learning gains, students’ prior experience, and the development of non-technical skills in order to better evaluate the effectiveness of virtual reality-based simulation training.

## Conclusions

Our findings indicate that simulation training is an engaging method for students to learn how to surgically remove a displaced wisdom tooth. Our results reveal that conventional simulation is subjectively perceived as superior to VR simulation by dental students, particularly in terms of realism. Moreover, VR simulation allows for a more objective evaluation of performance and outperforms the phantom model in terms of sustainability. At present, VR training cannot fully replace conventional phantom model training without limitations. However, further technological advancements and a more realistic VR representation could play a crucial role in maximizing the benefits of this innovative teaching method. Specifically, more precise calibration and better manoeuvrability are needed, along with adjustable parameters such as the instrument’s tilt angle and the patient’s head position. Additionally, the ability to practice incision techniques and local anesthesia administration could be integrated. Increased training time, extended briefing sessions and additional guidance on simulator handling would further support its implementation. A more realistic and more intuitive VR system could significantly contribute to leveraging the innovative advantages of this teaching method.

## References

[1] Gilbody J, Prasthofer AW, Ho K, Costa ML (2011). The use and effectiveness of cadaveric workshops in higher surgical training: a systematic review. Ann R Coll Surg Engl.

[2] Miller D, Crandall C, Washington C, McLaughlin S (2012). Improving teamwork and communication in trauma care through in situ simulations. Acad Emerg Med.

[3] Lu MY, Peng CY, Chang YC (2022). Interns’ perception of haptic virtual reality oral surgery simulator learning for impacted lower third molar extraction. J Dent Sci.

[4] Lehmann KS, Grone J, Lauscher JC, Ritz JP, Holmer C, Pohlen U (2012). Simulation training in surgical education - application of virtual reality laparoscopic simulators in a surgical skills course. Zentralbl Chir.

[5] Approbationsordnung für Zahnärzte und Zahnärztinnen (ZApprO) (BGBl. I S. 933), die zuletzt durch Artikel 4 der Verordnung vom 7. Juni 2023 (BGBl. 2023 I Nr. 148) geändert worden ist [Online]. ..

[6] Abschlussbericht VRmed - Virtual Reality in der medizinischen Lehre [Online]. ..

[7] Kuhn S, Huettl F, Deutsch K, Kirchgassner E, Huber T, Kneist W (2021). Surgical education in the digital age - virtual reality, augmented reality and robotics in the medical school. Zentralbl Chir.

[8] Waldmann Hcv UM, Stracke S, Fassnacht U, Gensichen J, Sönnichsen A, Öchsner W (2006). Review of patient simulation software - background, options and integration in medical education. ZFA (Stuttgart).

[9] Kröplin JHT, Geis C, Braun B, Fritz T (2022). eSurgery–digital transformation in surgery, surgical education andtraining: survey analysis of the status quo in Germany. Eur Surg.

[10] Li Y, Ye H, Ye F, Liu Y, Lv L, Zhang P (2021). The current situation and future prospects of simulators in dental education. J Med Internet Res.

[11] Higgins D, Hayes M, Taylor J, Wallace J (2016). A scoping review of simulation-based dental education. MedEdPublish.

[12] Roy E, Bakr MM, George R (2017). The need for virtual reality simulators in dental education: a review. Saudi Dent J.

[13] Murbay S, Neelakantan P, Chang JWW, Yeung S (2020). Evaluation of the introduction of a dental virtual simulator on the performance of undergraduate dental students in the pre-clinical operative dentistry course. Eur J Dent Educ.

[14] Buchbender M, Maser M, Neukam FW, Kesting MR, Attia S, Schmitt CM (2021). Kobra surgery simulator-A possibility to improve digital teaching? A case-control study. Int J Environ Res Public Health.

[15] St Pierre M, Breuer G (2018). Simulation in der Medizin Grundlegende Konzepte - Klinische Anwendung.

[16] ..

[17] ..

[18] Ayoub A, Pulijala Y (2019). The application of virtual reality and augmented reality in oral & maxillofacial surgery. BMC Oral Health.

[19] Towers A, Field J, Stokes C, Maddock S, Martin N (2019). A scoping review of the use and application of virtual reality in pre-clinical dental education. Br Dent J.

[20] Kroplin J, Maier L, Lenz JH, Romeike B (2024). Knowledge transfer and networking upon implementation of a transdisciplinary digital health curriculum in a unique digital health training culture: prospective analysis. JMIR Med Educ.

[21] Hsu MH, Yang HW, Chang YC (2022). Perspectives on the implementation of haptic virtual reality simulator into dental curriculum. J Dent Sci.

[22] ..

[23] Kanwal L, Gulzar M, Idrees W, Ikram F, Sukhia RH, Fida M (2024). The application of virtual reality and augmented reality in dentistry - a literature review. J Pak Med Assoc.

[24] Pohlenz P, Grobe A, Petersik A, von Sternberg N, Pflesser B, Pommert A (2010). Virtual dental surgery as a new educational tool in dental school. J Craniomaxillofac Surg.

[25] The L (2017). Artificial intelligence in health care: within touching distance. Lancet.

[26] Winkler-Schwartz A, Bissonnette V, Mirchi N, Ponnudurai N, Yilmaz R, Ledwos N (2019). Artificial intelligence in medical education: best practices using machine learning to assess surgical expertise in virtual reality simulation. J Surg Educ.

[27] van Gaalen AEJ, Brouwer J, Schönrock-Adema J, Bouwkamp-Timmer T, Jaarsma ADC, Georgiadis JR (2021). Gamification of health professions education: a systematic review. Adv Health Sci Educ Theory Pract.

[28] Rosser JC, Lynch PJ, Cuddihy L, Gentile DA, Klonsky J, Merrell R (2007). The impact of video games on training surgeons in the 21st century. Arch Surg.

